# Supplementary material for: Comparing and assessing four AI chatbots’ competence in economics
Source: PLoS One. 2024 May 8;19(5):e0297804. doi: 10.1371/journal.pone.0297804 (PMC11078351; doi:10.1371/journal.pone.0297804)
Supplement: S1 Appendix — (DOCX) [file pone.0297804.s001.docx]

|  | Bloom’s Taxonomy: **Analyze** | Bloom’s Taxonomy: **Evaluate** |
| --- | --- | --- |
| Microeconomics:  **Demand/Supply** | In the country of Wakanda there are many consumers with demand given by Q = 300 – 20p and many firms with supply given by Q = -100 + 80p. What will be the impact on consumer surplus if the government implemented a per unit tax of 2 paid by producers? | Economists have estimated that the price elasticity of demand for soda is -1.37 and currently the price of a soda is approximately 60 cents. At the same time, the medical community have estimated that reducing soda consumption by one 12 fl oz can per day lead to an average weight loss of 15 pounds, which in turn reduced the probability of diabetes by 2%. Using the information provided, evaluate the likely success of a 1.5 cent-per-ounce tax on soda. |
| Microeconomics:  **Firm Behavior** | Suppose a perfectly competitive firm produce candy bars according to the Cobb-Douglas production function, $Q={AK}^{0.5}L^{0.5}$, where A is 50. Suppose that capital is fixed at 100 units in the short run. Assume further that the price of capital is $50, and the price of labor is $15. If the market price of candy bars is $2, how many units of labor should the firm hire in order to maximize its profit? | Antitrust law is thought to give consumers several benefits. When one company isn't allowed to control the market, the public can shop and compare, which lead to lower prices and more variety of goods and services. A famous historical example of antitrust law is Rockefeller's Standard Oil, which in the late 19^th^ century dropped prices by more than 50% and bought up several of its competitors. As its control of the market increased, the company lowered production costs and the price of oil facing consumers dropped even more. In 1911, the government dissolved Rockefeller’s Standard Oil company into 43 smaller companies. Was this a good idea? |
| Microeconomics:  **Externality** | Consider the competitive market for paper. The inverse supply of paper is given by p = 10 + 0.015Q and the inverse demand for paper is given by p = 110 – 0.025Q. The production of paper also generates pollution and the total cost to society is $C = 0.01Q^{2}$. What is the optimal pollution tax and how much greater will total surplus be after this tax? | Consider two ways of protecting elephants from poachers in African countries. In one approach, the government sets up enormous national parks that have sufficient habitat for elephants to thrive and forbids all local people to enter the parks or to injure either the elephants or their habitat in any way. In a second approach, the government sets up national parks and designates 10 villages around the edges of the park as official tourist centers that become places where tourists can stay and bases for guided tours inside the national park. Consider the different incentives of local villagers—who often are living in poverty—in each of these plans. Which plan seems more likely to help the elephant population? |
| Macroeconomics:  **Inflation** | The total price of purchasing a basket of goods in the United Kingdom over four years is: year 1=£940, year 2=£970, year 3=£1000, and year 4=£1070. Calculate two price indices, one using year 1 as the base year (set equal to 100) and the other using year 4 as the base year (set equal to 100). Then, calculate the inflation rate based on the first price index. If you had used the other price index, would you get a different inflation rate? If you are unsure, do the calculation and find out. | Pat is ecstatic as they have just won the Powerball lottery game. The Powerball game has two options for Pat, they can either accept a lump sum cash prize which is 70% of the jackpot or an annuity paid in 30 installments of 29 years. Each installment is 5% of the total winnings after taxes. Suppose the tax rate is 25% and total winning are $60 million. Suppose the expected rate of inflation is 3% per year. Evaluate Pat’s two option and give a recommendation. |
| Macroeconomics:  **AD/AS** | The table below gives aggregate demand (AD) and aggregate supply (AS) for the country of Wakanda.   1. Plot the AD/AS diagram from the data and identify the equilibrium. 2. Suppose there is a tax cut that increases aggregate demand by 50 at every price level. Draw this change and identify the new equilibrium. 3. How will the new equilibrium change output? How will it change the price level? What do you think will happen to the rate of unemployment?  \| **Price Level** \| **AD** \| **AS** \| \| --- \| --- \| --- \| \| 110 \| 700 \| 600 \| \| 120 \| 690 \| 640 \| \| 130 \| 680 \| 680 \| \| 140 \| 670 \| 720 \| \| 150 \| 660 \| 740 \| \| 160 \| 650 \| 760 \| \| 170 \| 640 \| 770 \| \|  \|  \|  \| | Consider the aggregate demand (AD) and aggregate supply (AS) framework. If the economy is currently at long-run equilibrium and the natural rate of unemployment is 4.5%. What will happen to the rate of inflation and the unemployment rate if there is economic growth of 2% per year due to technological progress? What type of monetary policy would you recommend? |
